# The intersection between transgender identity and migrant background: Experienced barriers and facilitators to healthcare in The Netherlands

**DOI:** 10.1080/26895269.2024.2411533

**Published:** 2024-10-06

**Authors:** Eline Wijstma, Alex von Vaupel-Klein, Camiel Welling, Ali Jawad, Luella W. Smith, Raagini Bora, Sabrina Sanchez, Joël Illidge, Udi Davidovich, Elske Hoornenborg, Hanne Zimmermann

**Affiliations:** a Department of Infectious Diseases, Public Health Service of Amsterdam, Amsterdam, The Netherlands; b Trans United Europe, Amsterdam, The Netherlands; c Colored Qollective, Utrecht, The Netherlands; d European Sex Workers’ Rights Alliance, Amsterdam, The Netherlands; e Department of Social Psychology, University of Amsterdam, Amsterdam, The Netherlands; f Department of Work and Social Psychology, Maastricht University, Maastricht, The Netherlands

**Keywords:** Barriers to healthcare, facilitators to healthcare, intersectionality, migrants, qualitative

## Abstract

**Background:**

Transgender and gender-diverse (TGD) people, particularly those with migrant backgrounds (mTGD), face health inequalities and barriers to care globally.

**Aim:**

Examine barriers and facilitators to accessing and continuing healthcare within and beyond gender-specialist centers among mTGD people in the Netherlands.

**Methods:**

TGD community researchers conducted in-depth interviews with 32 mTGD individuals from twenty-seven countries. Interviews were inductively thematically analyzed.

**Results:**

Healthcare barriers relating to being TGD and being migrant compounded and interacted. Unmet transition needs, low socioeconomic status, and mental health issues were prevalent, and affected whether healthcare was a priority and access to healthcare. Having a supportive social network facilitated healthcare access. Beyond life circumstances, participants reported that providers outside gender-specialist settings had insufficient knowledge about TGD-related healthcare, and newcomers had difficulty bridging these gaps. The anticipation and experience of discrimination based on any facet of a participant’s identity formed a healthcare barrier, while identity validation was highly meaningful for participants and facilitated trust in providers. Continuity of caregivers was important to avoid having to re-explain one’s identity and needs, but alternating providers was often the reality - especially in refugee settings. Care models with TGD staff facilitated trust, and the integrated health and social care helped participants access healthcare whilst navigating complex life circumstances.

**Discussion:**

mTGD individuals face unique barriers to accessing healthcare owing to their intersecting migrant and gender identities. Key improvements are enhancing cultural competency and knowledge on TGD healthcare among providers, ensuring equitable access to GAMC for migrants, and integrating gender-affirming care with other healthcare and social support.

## Introduction

Health disparities are preventable differences in health and disease between social groups (Braveman, 2014). Communities that are marginalized often experience poorer health outcomes resulting from their discrimination, stigmatization, and exclusion in work, housing, healthcare, and social settings. Such circumstances leave individuals to be disproportionately exposed to stress which can lead to poorer health, and could lead to situations and behaviors that further compromise their health and wellbeing (Winter et al., 2016). On top of this, discrimination in healthcare settings can lead to healthcare avoidance by patients and delayed referral and diagnosis by providers (Hamed et al., 2022; Jaffee et al., 2016)

Communities with known health disparities include transgender and gender diverse (TGD) people and migrants. Previous studies demonstrated increased rates of cancer, cardiovascular health issues, HIV and other sexually transmitted infections, substance misuse and mental health issues among TGD and migrant communities (Connolly & Gilchrist, 2020; Leone et al., 2023; Pinna et al., 2022; Streed et al., 2021; Van Gerwen et al., 2020). Meanwhile, TGD and migrant individuals also face barriers to healthcare. Prior studies among primarily non-migrant TGD individuals described anticipated discrimination, insufficient provider knowledge on TGD-specific health outcomes, and culturally insensitive interactions as barriers to healthcare (Gieles et al., 2023; Hostetter et al., 2022; Kcomt et al., 2020; Wall et al., 2023). A systematic review summarizing studies among primarily cisgender migrant individuals described low health system knowledge among newcomers, language barriers, and low trust in providers as barriers to healthcare (Brandenberger et al., 2019).

In 2017, it was estimated that 48,000 individuals in the Netherlands were transgender, and that a quarter of those individuals was also migrant (Government of the Netherlands, 2018; Sociaal Cultureel Planbureau, 2017). According to Kimberlé Crenshaw’s theory of intersectionality, their lived experiences are formed by the unique intersection of their migrant and TGD identity, and can therefore not be inferred solely by combining the experiences of cisgender migrants and non-migrant TGD individuals. While some studies described healthcare experiences among TGD people who were black, indigenous or people of color (BIPOC) (Howard et al., 2019; Sherman et al., 2022), to the best of our knowledge none focused specifically on migrant TGD (mTGD) individuals (i.e. individuals not born or raised in the country in which they seek healthcare). Therefore, this qualitative study examined the experienced barriers and facilitators to accessing and continuing healthcare for various health needs among mTGD people living in the Netherlands.

## Methods

This inductive qualitative study used principles of community-based participatory research (CBPR) to involve community members throughout the research process (Israel et al., 1998). The study team included mTGD community members with and without prior research experience. They co-designed the interview guide, conducted the in-depth interviews, and provided input on the results synthesis and feedback on this manuscript.

### Setting and participants

Eligible participants were identified from the visitors list of the Amsterdam Trans Clinic. This clinic is a collaboration between the Center for Sexual Health of the Public Health Service of Amsterdam and Trans United Europe, a community organization working to empower the BIPOC trans community and operating from Amsterdam. The Amsterdam Trans Clinic offers integrated hormone replacement therapy (HRT), sexual healthcare, and psychosocial support to TGD individuals ≥18 years, and is specifically aimed at TGD individuals who belong to multiple marginalized communities. For example, those who are also migrant (including refugees, asylum seekers, and undocumented persons), uninsured, unhoused or staying in a crisis shelter, engaged in sex work, or who experience exclusion based on skin color, ethnicity, language barriers, or religion. Visitors of the Amsterdam Trans Clinic were eligible for study inclusion if they were born or grew up outside the Netherlands, had sufficient command of the Dutch or English language, and provided informed consent for study participation. We used purposive maximum variation sampling to recruit people with varying ethnicities, genders, ages, migration histories, legal statuses and educational backgrounds (Green & Thorogood 2018).

### Procedures

Interested and eligible participants were linked to an interviewer (AJ, ESJ, RB, SS, AvV) to whom they had no personal or professional relationship that could hinder perceived confidentiality or candidness. Interviews took place between July 2021 and August 2023 at a community safe space, the Public Health Service of Amsterdam, or online *via* MS Teams or Zoom based on participants’ preference. Travel costs to and from interviews were covered, and participants were offered a €20 gift voucher for participation.

Interviews followed a semi-structured interview guide and lasted approximately one hour. The guide comprised questions regarding participants’ experiences of locating, accessing, and continuing healthcare for any health need and suggestions for improvements (Appendix A). Examples of questions include: “What type of healthcare have you needed?”, “How did you go about getting that healthcare”, “Were there any obstacles or helpful factors that you encountered while trying to access that healthcare?”, and “In what way did factor X make it more difficult/easier to access the healthcare you needed?” Interviews were audio-recorded, and were transcribed verbatim by AvV, EW, HZ, or an external transcription agency with whom the Public Health Service of Amsterdam has a longstanding confidentiality agreement. Names and other identifiers were censored in transcripts. Audio recordings will be deleted after publication of the manuscript.

After the interview, participants completed a short demographic questionnaire (Appendix B).

We included new participants until reaching data saturation.

### Data analysis

We used MAXQDA Plus 2022 software (Verbi GmbH, Berlin) and followed Braun and Clarke’s six phases of thematic analysis (Braun & Clarke 2006). First, getting familiar with the data: AvV, EW, and HZ transcribed and read the first six interviews. Second, generating initial codes: AvV and EW independently open-coded the first six interviews, and discussed labels until reaching consensus. Third, searching for themes: AvV and EW grouped labels into categories, and categories into themes. Fourth, reviewing themes: themes were reviewed whilst analyzing new interviews; prior interviews were iteratively re-coded if applicable (AvV, EW). Themes were also reviewed during three separate discussions with the full research team. Fifth, defining and naming themes: we defined major themes with the full research team. Sixth, producing the report: one researcher (EW) produced the draft manuscript, during which she re-read all interviews to ensure the report stayed close to the data. All team members provided feedback on the manuscript.

### Ethical considerations

The Amsterdam University Medical Center Ethics Committee deemed this study exempt from ethical approval by an institutional review board, since procedures do not infringe on participants’ physical or psychological integrity (Central Committee on Research Involving Human Subjects (CCMO)). Informed consent to was obtained from all individual participants included in the study, on audio-record. We clearly communicated that the decision about participating would in no way affect a patient’s access to services of the Amsterdam Trans Clinic. Participants could withdraw study participation at any moment.

### Positionality of the research team

To position ourselves in relation to this work, we disclose the aspects of our identities that are relevant to this project. Of the 11 members of the research team, 8 are part of the LGBTQI+ community, of whom 4 are TGD individuals. Seven members of the research team grew up in or have parents from outside of Western Europe, representing a variety of regions in the world, including Latin America, North America, the Caribbean, South Asia and the Middle East. Team members have educational backgrounds in medicine (AvVK, CW, EH), psychology (AJ, UD, HZ), health sciences (EW, ESJ, HZ), medical anthropology (JI), and sociology including gender studies (AvV, JI, RB). Five team members (CW, SS, ESJ, RB, JI) were on the board of organizations advocating the rights of LGBTQI+ individuals while this research was being conducted.

## Results

Table 1 presents the characteristics of the 32 study participants at the time of their interview. 18 identified as (trans) woman, 8 as (trans) man, and 6 had another gender identity. Ages ranged between 19 and 47 years. Twenty-seven unique birth countries spanned 7 world regions. The majority of participants started college- or university-education (*n* = 21, 65.6%). Less than half (*n* = 15, 46.9%) cited formal work as their main source of income.

**Table 1.** Participant characteristics at the time of their interview.

		Total (*n* = 32)	
Variable		n	(%)
**Gender identity**	(Trans) woman	18	(56%)
	(Trans) man	8	(25%)
	Non-binary*	6	(19%)
**Sex assigned at birth**	Male	18	(56%)
	Female	12	(38%)
	Missing	2	(6%)
**Age in years, median (range)**	** **	30	(19–47)
**Age category in years**	19–24	7	(22%)
	25–29	8	(25%)
	30–34	9	(28%)
	≥ 35	8	(25%)
**Region of birth**	Latin America and the Caribbean	9	(28%)
	Eastern/Central Europe and Central Asia	8	(25%)
	Middle East and North Africa	6	(19%)
	East Asia and the Pacific	3	(9%)
	South Asia	2	(6%)
	Sub-Saharan Africa	2	(6%)
	North America	2	(6%)
**Highest started education**	College / university	22	(69%)
	Practical school / trade school	1	(3%)
	High school	7	(22%)
	Missing	2	(6%)
**Dutch residence status**	Dutch / EU citizenship	12	(39%)
	Residence permit	10	(32%)
	Refugee status or in asylum procedure	6	(19%)
	Study or work visa	2	(6%)
	Undocumented	1	(3%)
	Missing	2	(6%)
**Health insurance**	Dutch health insurance	23	(72%)
	Asylum seekers’ health insurance	7	(22%)
	No insurance	2	(6%)
**Main source of income**	Formal work	15	(48%)
	Government support	14	(45%)
	Informal work	2	(6%)
	Financial support by family/friends/acquaintances	0	(0%)
	Missing	1	(3%)
**Housing situation**	Independent housing	13	(41%)
	Shared housing	7	(22%)
	Staying with family/friends/acquaintances	6	(19%)
	Refugee center	5	(16%)
	Other (not specified)	1	(3%)

Abbreviations: EU: European Union; *Self-described non-binary gender identities included: non-binary (*n* = 2), both male and female (*n* = 1), neither male nor female (*n* = 2), both male and non-binary (*n* = 1)

**Figure 1.**
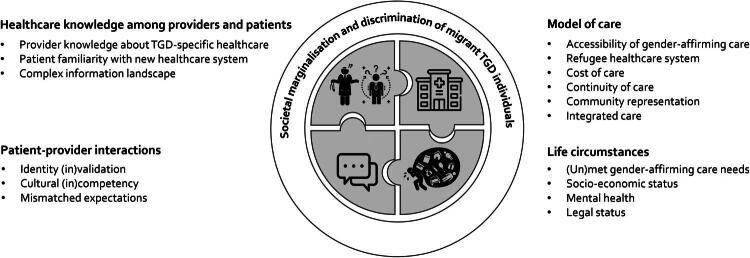
Explanatory conceptual framework comprising the main themes regarding experienced barriers and facilitators to healthcare for migrant transgender and gender diverse individuals.

We developed four main themes relating to healthcare access: knowledge among providers and patients, patient-provider interactions, model of care, and life circumstances (Figure 1). Themes related to healthcare access in one or more of three ways: by impacting practical access, by impacting willingness to initiate or continue healthcare, or by impacting whether participants prioritized healthcare. Barriers relating to gender identity and to migrant identity compounded on and interacted with each other.

### Theme 1: Healthcare knowledge among providers and patients

Participants described that many providers outside gender-specialist settings appeared unfamiliar with trans-specific health needs or the landscape of gender-affirming medical care (GAMC). Some participants had the impression that their provider lacked the confidence to provide general healthcare to them when they were referred to a gender-specialist. Both situations formed a direct healthcare barrier (i.e. when participants were not assisted appropriately), and an indirect healthcare barrier by impeding trust and willingness to continue care. Insufficient provider knowledge formed the largest barrier for newcomers in the Netherlands, who had difficulty bridging providers’ knowledge gaps or misconceptions.

“As an international persons I am quite unfamiliar with how the Dutch [GAMC] system works. […] The second [issue] was that my GP didn’t understand the [GAMC] system either.” (P42)

#### Provider knowledge as direct barrier or facilitator

Thirteen participants reported as a barrier that their GP was familiar with one gender clinic in the Netherlands, but not other facilities providing care for TGD persons. This particularly affected participants with TGD-related healthcare needs beyond initiating HRT (for example, mental health support relating to their gender identity). They were sometimes needlessly placed on a waitlist for this gender clinic.

“A lot of general practitioners in the country only know about the gender clinic in Amsterdam, not the other gender clinics in the country.” (P76)

Insufficient knowledge of TGD-related mental healthcare among psychologists (mentioned by 7 participants) led to delayed treatment or treatment of symptoms without addressing gender incongruence as the root cause. Some participants stopped discussing gendered experiences with their psychologist after receiving *“inaccurate responses”.*

“When I went to mental health providers, they would often express their own limitations with dealing with trans patients and the adequate therapy to give. So I would often be sent to many different places.” (P81)

“When I was speaking to professionals and they know nothing about the gender dysphoria, it was depressing even more. I know that they are trying to help, but the antidepressants itself wouldn’t work.” (P76)

Several participants thought that their gender identity ‘distracted’ physicians from following general clinical reasoning: participants described unnecessary referrals to gender-specialist care, and the rapid misattribution of symptoms to their medical transition.

“Even you go and you say: ‘I have a headache’, they say: ‘O, I’m not specialized, I’m sorry’.” (P141)

“And I figured out for myself why I have high blood pressure. […] The doctor didn’t think about it [sleep apnea as a diagnosis]. She was just like: hormones must be it.” (P123)

It appeared that some providers were unaware of, or held misconceptions about healthcare options for refugees. For example, some refugee participants were told that it was impossible to get HRT before acquiring Dutch residence status, even though refugees have the right to access HRT if they used HRT before arriving to the Netherlands (Kennisinstituut van de Federatie Medisch Specialisten, 2024). Moreover, two undocumented participants were turned away by a GP or pharmacy, being told they could not get a prescription or medication. It was unclear whether the providers in question were unaware of the existence of medical services for undocumented people, or missed an opportunity to link to these services. As a result, one undocumented participant lived with concerns about their health.

“I had a document from my last asylum seekers center with prescription [for HRT], with a therapy. They [GP] can see it, but they refused to continue as I’m an undocumented person. They said: ‘We cannot prescribe you’” (P141)

“I take care of myself, because at the end of the day, I’m the back-up, I have nobody to fall on. […] I have to [take care of myself]. If I get sick tomorrow, what’s going to happen?” (P175)

Finally, one participant explained how her parents received one-sided information about transitioning from a GP and subsequently a psychologist. According to her parents, both healthcare professionals said that transition generally coincided with regret, depression, and suicide—whilst empirical evidence suggests that regret is uncommon (van der Loos et al., 2022; Wiepjes et al., 2018) and that transition generally improves life satisfaction (James et al., 2024). The misinformation exacerbated the existing gender-related struggles with her parents, and made her feel that these providers placed their “*opinion*” above their professionalism.

“When my family found out about the whole transitioning thing they went to their GP to ask about it, and their GP was like: ‘Oh yeah, transitioning is, these people do it, and they spend years in depression, and in the end they kill themselves. So it’s a good idea for me not to transition’. […] In turn, it basically meant that I get way less support from them or way more resistance from them to do stuff or to come out to them or be comfortable with them. So I think that’s the most frustrating thing. Even more than all the doctors and the waiting lists.” (P95)

#### Provider knowledge as indirect psychological barrier or facilitator

When providers lacked knowledge around healthcare for TGD patients, this also impacted trust in and feeling safe with providers. First, because participants found it tiring and burdensome to educate their providers on basic terms and concepts (e.g. pronouns, the difference between social and medical transition), correct misconceptions and stereotypes, and explain TGD healthcare guidelines.

“It’s a bit strange that the patient has to give them [GPs] that knowledge, and so they use my energy and my secureness of going there, teaching them. I’m in a position now where I could do it, but I’m also, I don’t really need them, because I have these other ways [to take care of my health].” (P118)

Second, some participants believed that their provider claimed to have insufficient knowledge to mask that they were unwilling to provide care to TGD individuals. This belief mainly arose when providers took no effort to learn about TGD healthcare, and *“distanced themselves”* from a participant’s health needs.

“I think [my GP] is just willfully ignorant. […] They pretend they don’t get it, because that’s a very good way of them exercising power. Because they don’t like you and they can just say: we don’t get it, we’re not competent enough, because what can I say?” (P121)

Good provider knowledge on TGD health(care) facilitated trust in providers. Trust was also facilitated when providers showed willingness to learn about TGD health(care), for example by looking up information on the spot.

“[My GP] is very nice and very welcome. I asked: do you have any information about the transitioning? And she told me that she doesn’t have any information at the moment, but if I wanted she can do a little bit of research and [she] tried to ask a colleague.” (P84)

#### Finding healthcare information as a newcomer

Several participants found it difficult to navigate the Dutch healthcare system as a newcomer. Specifically, they found it complicated to learn which healthcare facilities exist, how to arrange health insurance, or how to register at a GP practice.

“It was difficult. [.] You need to do everything yourself. You need to find a doctor, pharma. You need to go to look for people to help you and make contracts for the energy, contracts with the insurance. Yes, it was very difficult to find help.” (P75)

They explained that healthcare information was often dispersed across websites, unclear, or only available in Dutch. Participants not fluent in English had particular difficulty finding healthcare information. Online translation tools (e.g. Google Translate) were helpful, but some participants felt overwhelmed or discouraged by having to translate an already complex information landscape.

“Everything is in Dutch. So, it’s a little like, sort of like… You feel discouraged and you just feel like, OK, maybe I shouldn’t do this. Maybe I just postpone it [getting care]” (P61).

Having a social network was therefore the most frequently mentioned facilitator to getting healthcare-related knowledge and subsequently accessing healthcare (*n* = 16). Still, some participants—mostly men—avoided discussing healthcare with peers, especially stigmatized needs relating to sexual or mental health.

“I honestly think that if my friends didn’t tell me about the clinic, I wouldn’t have found the clinic, because as I’m not speaking Dutch at all. It’s not that easy to search online and find things. It’s just like, I don’t know, trying to find a needle in a really big space.” (P76)

“Ladies, they share.[…] But the guys don’t.” (P59)

Some participants found healthcare information through LGBTQI+ -focused social media accounts or dating apps. A reported downside was that you would need prior knowledge on which accounts to follow.

“If we don’t know that the organization exists, then we don’t know where to search.” (P76)

To improve health literacy among mTGD, several participants suggested that LGBTQI+ and migrant-focused organizations perform more outreach activities to share healthcare information.

“For trans people for better able to get services, [it] is not just social media. You [i.e., healthcare organizations] have to go out. You have to make a visibility in person, in the streets.” (P7)

### Theme 2: Patient-provider interactions

Trust in healthcare providers was vital to participants’ decision to seek or continue healthcare, especially in settings where they had to disclose their trans identity, discuss stigmatized health needs, or undergo physical examination. Participants’ trust was influenced by several things. First, participants’ pre-conceived notions about LGBTQI+-acceptance among healthcare providers (which was in part affected by interactions with other authorities like the police and security guards in the refugee center). Second, stories from their social network (“*people don’t trust [doctors], because they hear a lot of bad things”,* P141*)*. Third, their own prior patient-provider interactions. We identified kindness and empathy, identity (in)validation, objectification, and cultural differences as main factors which affected trust in healthcare providers. Importantly, some (*n* = 10) participants attributed negative patient-provider interactions to assumed transphobia or other discriminatory attitudes from their provider. Most found it difficult to explain precisely why they suspected such discriminatory attitudes. Some participants felt that their intersectional identity made them vulnerable to constant discrimination on multiple axes.

“[Medical professionals] are not exposing their transphobia or homophobia openly, but you can see it, you can feel it with the body language, with the ignorance also. […] It’s quite difficult to express or explain. You just have to have [experienced] it and know it.” (P141)

"I’m going through doctors experiences, because sometimes it’s racism, sometimes it’s misgendering, sometimes it’s like, even if I get a person of color, they may not be racist, but they might be transphobic." (P123)

#### Kindness and empathy

An important facilitator to healthcare access was kindness and empathy from providers toward participants, as it made them feel safe to visit healthcare and discuss their health needs. For some, a simple display of kindness was highly meaningful because it formed a much-needed contrast to the discrimination that they often faced in society. Three participants described their kind and empathetic provider as ‘family’.

“With her [i.e., GP], I can actually share everything. She’s very nice and very welcome.” (P84)

“Even just that small amount, some kindness goes so far. Because I receive no fucking kindness.” (P60)

#### Identity validation

Many participants said that identity validation from healthcare providers was vital to trust: “*validation is the core of our happy existence*” (P15). We identified the following provider practices relating to patient identity validation.

Explicit dismissal of a patient’s gender identity (e.g. one female participant was told: *“you are a man”*) was a barrier, whereas gender affirmation was a facilitator.

Misgendering (i.e. addressing someone with the incorrect pronouns or titles) and deadnaming (i.e. addressing someone by their birth name instead of chosen name) were barriers. Misgendering and deadnaming impeded trust especially when it occurred systematically or when participants felt it was easily avoidable (e.g. when chosen name and pronouns were available on record). It was a facilitator when providers actively asked, registered, and used a patient’s preferred pronouns.

“In the first few weeks, she [the psychologist’s assistant] kept misgendering me. And every time I corrected her and then at one point I went to appointment ready to say like: I’m not going to have any more appointments with you, because you clearly don’t respect my identity, therefore you don’t respect me.” (P11)

“During our first days together she [GP] actually changed my name in the system into the name that I usually use or I like, which made it easier for us to communicate. Because if she mentioned me first legal name I would probably freak out.” (P76)

It was a barrier when providers referred to post-operative body parts as “fake”, or contrasted trans men/women to “real” men/women. Trans-inclusive language use was a facilitator.

“The way they said it, was that my vagina was fake or something of this sort. […]

And it kind of made me feel like I didn’t want to go anymore.” (P61)

“[The GP] told me: ‘Even women don’t have this kind of fat [breast] milk’, which was quite insulting for me. What do you mean ‘even women’?” (P141, (trans) woman)

Unprompted comments on a patient’s transition journey formed a barrier. The following participant lost trust in her GP after he got angry that she formally started HRT.

“He [GP] was angry. Like, ‘yeah, you shouldn’t started [HRT]’. […]I lost trust. Even like, I have toothache, or anything, I wouldn’t, I didn’t go. […] And when medically there is something wrong, mostly I ignore it.” (P15)

It was a facilitator when providers made patients feel seen as a person beyond their transgender identity.

“I just want to be seen as a human being who needs help. Having someone there to actually provide that was amazing for me.” (P11)

“When I bring up any of my issues, they are seldom trans-related. […] It’s just common human problems. […] To see me as a person is the most helpful thing there is.” (P58)

#### Objectification, sexualization, and misconduct

A number of participants had felt objectified in healthcare settings, with some feeling like an *“alien”, “science experiment”*, or *“lab rat”*. These feelings emerged after their provider responded awkwardly to their gender disclosure, or asked irrelevant questions about their body or transition. Sometimes, objectification included sexualization. Multiple participants were asked invasive questions about their sexual life outside sexual healthcare settings, which they attributed to the societal stereotypes about TGD people.

“I do feel like they [doctors] assume that it [i.e., being TGD and sexual risk behavior] is linked, based on the questions they bring up, that I’ve never heard in any other medical context being asked.” (P81)

Two women experienced sexual misconduct by a physician, in which they felt their physician acted on curiosity around trans bodies. One participant described how her physician in the refugee center asked to see her neovagina as proof of her transness, before continuing her hormone support. The other participant described the way in which her GP touched her breasts and took a breast milk sample during a breast cancer screening.

“I asked [the physician]: ‘can you please give me the hormones?’ […] He said: ‘Okay, can I see your vagina?’ […] And then after that he touched my pussy.” (P199)

“I feel some personal things sexualising the conversation and the way he was looking at me, from the first visit. But the second [time] when I visited him with the complaints of breast, he just took the opportunity to touch me. […] The way he was doing it, it was not a physical check.” (P141)

Both women described their encounter as traumatic, but neither reported it to authorities. They cited the following reasons: their marginalized legal status (i.e. refugee/undocumented person), lack of knowledge about the Dutch healthcare system, and fear for deportation.

“I feel not good [to report medical misconduct], because I’m here and I’m a transgender women and I’m here in refugee. But I don’t understand why he checked my pussy. Okay, I don’t know in Netherlands articles, but the doctor knows in Netherland articles. The doctor knows the Netherlands’s rules.” (P199)

“At that time I was not documented and illegal. I will of course complain and something, but I was trying to avoid any kind of legal problems to not be deported, so that was a reason why I just calmed myself down and say: okay, maybe this happened, no worries, I’m still alive, nobody beats me or something.” (P141)

#### Language barriers and cultural differences

Some participants were not proficient in English nor Dutch. This resulted in difficulty communicating health needs to providers and misinterpretation of the received health-related information and tone of conversation (e.g. more often perceived as insensitive).

“You need to explain: what do you feel, what do you want, what is difficult for you at this moment? […] That was difficult because my English is not very good and clear. I had problems with expression. […] If you don’t speak English, you are lost.” (P75)

Mismatched expectations of care due to cultural differences also formed a barrier. Many participants felt that Dutch GPs were unhelpful because Dutch GPs were more conservative to prescribe medication or refer to other providers compared to GPs in their country of origin.

“Let me just be clear and straight why I don’t go to the GP. You’ll be sick, really sick and they will tell you: come tomorrow. […] Then you’ll give me Paracetamol.” (P59)

Some participants interpreted their provider’s attempt at shared decision-making as placing the burden of diagnosis and treatment on the patient.

“Every time I went to [the GP], she’s like: ‘How do you think, what do you think it is?’. […] So I feel it’s really not useful. I'm not sure, maybe that’s a culture difference. Because back in my country, we expect the doctor to know, or research, rather than the patient do their own research.” (P158)

Finally, two participants felt that Dutch providers spoke too loudly, thereby impeding on privacy.

“Especially at the receptions, medical receptions, all over Holland. [Staff speaks] too loud. […] Because coming from a society that it’s so taboo, where everybody is really concerned [for their privacy].” (P7)

### Theme 3: Model of care

#### Model of gender-affirming medical care

All participants reported systemic barriers that directly impacted their continuation or access to GAMC upon relocation to the Netherlands. Main barriers were the extensive waiting lists for GAMC and the ‘diagnosis model’ of transgender care (where a formal psychiatric diagnosis of gender dysphoria by a gender specialist is required to access HRT or gender-affirming surgery (Coleman et al., 2022)). Generally, participants described the diagnosis model as “*pathologizing”*, “*dehumanizing”* and exclusionary of atypical gender experiences. Nineteen participants voiced a preference for the ‘informed consent model’ of transgender care (where self-determination about gender identity determines eligibility for HRT or gender-affirming surgeries (Gerritse et al., 2021)). Participants found the ‘informed consent model’ to be empowering, and a facilitator of trust and honesty toward GAMC providers. This system was also believed to facilitate a shorter time until HRT initiation.

“They wanted to prove if really I’m trans and I say: ‘Please, I’ve known myself ever since I was a child, I’ve never lived another life than the life that I’m living right now.’” (P59)

#### Continuity of care

Eight participants who had previously been assessed for gender incongruence, started HRT, and/or had gender-affirming surgery in their country of origin, were placed on a multiple-year-long waiting list to be re-assessed for gender incongruence in the Netherlands. In contrast, participants who used chronic medication for HIV or diabetes said that their medication was continued successfully and with ease upon arrival to the Netherlands.

“I had to explain my whole story, my discomfort or dysphoria and, eh, it takes so long. I already have hormones for years now and had my mastectomy. They did want to hurry up the process, but it still had to go through all the consultations” (P118)

Frequent rotation of providers was mentioned as a barrier in the context of GAMC (*n* = 4), psychological care (*n* = 5), and general healthcare (*n* = 2). With each new provider, participants had to re-explain their gender identity and transition history, face the possibility of discrimination, and re-build trust. In some cases, participants needed to re-justify their gender-related health needs and felt they had to reeducate their provider on TGD health.

“The GP where I am registered, it’s basically, you get whichever doctor is free. So, the first time I went there, I spoke to this woman. […] She was very kind and understanding. […] And then I spoke to a man, and he was like, oh okay. Like, the energy just shifted [after disclosing my trans identity]. […] So, the third time, I met another man, and he was calm about it [my gender identity]. The only issue he had was not misgendering me.” (P58)

#### Model of refugee healthcare

Twelve participants mentioned that they arrived in the Netherlands as asylum seeker. In the refugee healthcare system, some did not get clinically-indicated specialist care due to conservative referral policies. Some could not access HRT because the provider who was available in the refugee center was unwilling to provide HRT.

“Soon I will need to [have] a prescription to get new [HRT] medication. She [i.e., a fellow transgender resident in the refugee center] said: ‘It is going to be not possible for you here’. […] If you are not giving any other choice to asylum seekers to have another insurance or another organization, if it’s only you, then do it good.” (P141)

Rotation of providers frequently occurred within the refugee healthcare system. Because healthcare for refugees is linked to their center of residence, they get new providers whenever they move centers. Regrettably, two participants who struggled with rotating providers in the refugee healthcare system mentioned that gender-based harrassment from fellow refugee center residents was the reason they had to move.

“When you move from a [refugee] center to another, the psychologists switch. But I need to build a relationship of trust between me and this person that I’m going to tell exactly how I got raped when I was [age] years old.” (P15)

#### Community-based care, peer-led care, and explicit LGBTQI+-inclusivity

About half of participants voiced a preference for community-based or peer-led care over traditional care settings because it feels safe and comfortable to receive care from fellow LGBTQI+ persons. Some furthermore felt that fellow trans or queer providers were less prone to essentialize them to their gender identity, and could therefore better respond to their healthcare needs (see ‘Patient-provider interactions’ section). Community-based care settings were also appreciated for helping participants accept themselves and build a social network of peers.

“You get to feel the sense of like: this [clinic] is made by trans people and queer people and people of the community. They know that, you know, how we’re presented on TV and how we’re presented in like, outdate decades of research is not actually representative of how we are as people. Yes, I don’t feel like a little science experiment. I felt like a person.” (P60)

“I arrived here to [the clinic] through a trans friend. She explained to me that at least here I could find a doctor. Even if I couldn’t find a doctor, I could find a community and support from people from my own community, which is something I never had before.” (P15)

Some participants intentionally searched for a trans-inclusive GP or psychologist, in order to enter care with confidence. In their search, they used an online registry of LGBTQ+-friendly healthcare providers (e.g. www.rozeinwit.nl) or took notice of pictures, statements, or symbols on the websites or social media of healthcare institutions.

“There is a website that has all the trans and gay friendly GPs, and the one I went to was on the list.” (P158)

Finally, participants suggested that healthcare institutions should hire TGD and migrant people at all levels of the system—from physicians to policy-makers—to sustainably improve inclusivity in healthcare.

“The fastest route [to improving healthcare for mTGD] is actually having people from that community partake in law-making and discussions, and having them in places where you hear their voices loudly and adequately” (P58)

#### Integrated care

Multiple participants suggested that integrated care systems (i.e. that provide multiple types of care alongside each other) could improve healthcare access for the mTGD community. This was because transitioning involved somatic, mental and legal aspects, and because many participants additionally faced precarious circumstances like mental health issues or low socio-economic status. Addressing all these concerns in separate settings was perceived as complicated and overwhelming, and not aiding toward “*the general wellness of LGBT refugees”* (P93).

“Transition by itself is a very, you know, complicated, uh, matter. Inside, outside, medically, emotionally, socially, even paperwork.” (P15)

“I have the endocrinologist, I have the person at [psychology clinic] […], and then there’s like another person for legal matters, and then there’s another person for therapy, and another person for surgeries. And none of them know each other. So the network of me as a patient is very much separate and all-over the place.” (P81)

### Theme 4: Life circumstances

Many participants experienced a multitude of precarious life circumstances, like low socioeconomic status, unmet transition needs, and mental health issues. These circumstances formed practical barriers to healthcare, coincided with anticipated discrimination in healthcare, and led to a de-prioritization of healthcare. The absence of these factors and the presence of a social support system were facilitators to healthcare access.

“So, transition, being trans, being refugee, housing problem. It’s a lot. It’s overwhelming.” (P15)

#### Socio-economic status

Many participants were in a precarious economic position at some point in life, which formed practical and mental barriers to care. Practical barriers included not being able to pay for health insurance, deductible excess costs, or travel to care facilities. The following participant, who became homeless after leaving the refugee center due to gender-based violence, explained how couch-surfing affected travel time to health facilities and subsequently access to healthcare. She furthermore mentioned how small reimbursements of travel costs helped her to access public healthcare.

*“Many times I felt ill or sick, and I didn’t go to doctor because I cannot travel two hours in the train while I'm sick.” (P15)*

“[The public health clinic] paid my costs to come from the [refugee] center [to the clinic], which is like, these little details, that matters! Because sometimes, without this little money to come, I cannot come.” (P15)

As a mental barrier, some participants in precarious economic circumstances chose to postpone care-seeking to a period with more life stability.

“Now I’m just waiting for my house and then after I focus on my life.” (P199)

#### Unmet transition needs

Unmet transition needs, which often resulted from barriers to GAMC or legal gender recognition had an evident impact on participants’ ability to thrive as their authentic selves. It also affected access to other forms of healthcare. First, because participants with unmet transition needs or in the process of transition had little mental space or time to engage with other, non-acute or preventative care, stating *“you can’t do everything”* (P118).

“[Facial feminization] is like my life priority. Like, even before eating, before going anywhere, before breathing I must do these surgeries, because, yes, it’s difficult without them. It’s impossible without them. [….] For me as a transgender [person] my face is more important than my heart.” (P73)

Second, some participants developed severe body dissatisfaction during periods without access to GAMC and therefore had difficulty leaving the house completely or to see a doctor.

“I don’t like to show my body to someone else. I don’t know if you can get it or not, but I’m like this. When I have no surgery I can’t go to the doctors or something. I just can’t. I don’t feel safe.” (P110)

Third, participants described how not ‘passing’ (i.e. being perceived as their self-identified gender, without recognition of their transgender status) as a result of unmet GAMC needs could jeopardize their safety in public spaces and healthcare settings.

“Coming to the [the clinic], I have to go through the [city] center. [.] I’m scared to walk there. […] Sometimes people spit at me, sometimes people say the ugliest words you can ever imagine. […] It’s really difficult. Just because I’m not passing.” (P73)

“When people can’t pin you down, that’s when they hate you the most.” (P60)

Multiple participants linked their unmet GAMC needs to their migrant background. For example, the impossibility of starting GAMC at a younger age in their country of origin, as well as their ethnic features, enhanced their need for services like laser hair removal or facial reconstructive surgery.

“My biggest issue as transgender [person] was the hair. […] Especially coming from the Middle-East, because Middle-Easterns are a little bit hairy. […] The cost of laser is so high and I cannot afford that of course, because I don’t work, I’m just living with alimony.” (P73)

#### Mental health concerns

Many participants dealt with mental health concerns relating to their gender incongruence or to traumatic events that occurred in their country of origin or the Netherlands. Twelve participants mentioned ever feeling depressed, 7 ever had suicidal thoughts, 6 reported anxiety, and 10 dealt with trauma. We identified three important implications. First, mental health concerns lowered the desire for good physical health and the ability to seek healthcare. Second, having both gender-related and gender-unrelated mental health needs complicated finding appropriate mental healthcare: often, each need was addressed by a separate provider. Third, prior traumas increased the need for safe healthcare environments and sensitive patient-provider interactions.

“I feel that the Dutch mental healthcare system is not equipped for people who have more than one issue. […] Not only am I trans, I also have like childhood trauma that needs to be resolved. I also am, I have ADHD and I have performance anxiety.” (P123)

#### Social network

Having a social network facilitated healthcare access beyond information-sharing. Friends also encouraged care-seeking, functioned as interpreters, or assisted participants who did not feel comfortable going to a health provider on their own. Friends also supported participants through other precarious circumstances like periods of poor mental health and socioeconomic instability.

“I became homeless because I could no longer pay my rent, and some friends took me in. […] They got me the psychological help I needed. They helped me come out of the closet” (P11)

However, some participants had difficulty building a social network. For example, because they were new in the Netherlands or because their refugee center was located far from LGBTQI+ community hubs. The availability of LGBTQ+ community spaces (including spaces specifically for migrants and refugees) was therefore cited as important facilitator to healthcare access.

“I live in a city where there is not much activities for the queer community, which makes it hard to find people from the community. And for people who are not part of the community, sometimes there will be some awkward questions, which makes me avoid them.” (P76)

## Discussion

This study explored the barriers and facilitators to accessing and continuing healthcare for migrant TGD individuals in the Netherlands, as they navigate healthcare beyond gender-specialized facilities. Access to healthcare was impacted both directly through systemic barriers and indirectly through factors which affected willingness to seek or continue care. We developed four main themes. First, insufficient provider knowledge around TGD-related healthcare formed a practical and mental barrier to care, especially for newcomers who also had limited health system knowledge. Second, patient-provider interactions positively or negatively affected participants’ trust in healthcare providers and thereby motivation to seek or continue care. Third, certain healthcare models (e.g. the refugee healthcare system and diagnosis model of transgender care) hindered healthcare access whereas others (e.g. integrated and community-based care systems) facilitated healthcare access. Fourth, precarious life circumstances like low socioeconomic status, mental health problems, and unmet GAMC needs formed practical and mental barriers to healthcare, whereas stable life circumstances and having a social network facilitated healthcare access.

Within each theme, we found elements that shared similarities with previous research that separately investigated barriers related to gender identity or to migrant background. Common themes between migrant and non-migrant TGD people were: insufficient provider knowledge to provide care for TGD individuals, gender-based discrimination and stereotyping, trans-exclusive language, unmet GAMC needs, and low economic status (Gieles et al., 2023; Hostetter et al., 2022; Kcomt et al., 2020; Kommattam et al., 2017; Wall et al., 2023). Shared themes between cisgender migrant and TGD migrant people were: discontinuity of previously initiated care, limited insurance coverage for refugees, unfamiliarity with the healthcare system, language barriers, racism, mismatched cultural expectations of healthcare, and low economic status (Brandenberger et al., 2019; Khanom et al., 2021). In our study, we found that gender- and migrant-related barriers compounded (i.e. ‘additive disadvantage’) and interacted (i.e. ‘intersectional disadvantage’), illustrating the unique challenges and needs of the mTGD community when trying to access healthcare.

Consistent with prior quantitative studies among TGD persons (Grant et al., 2011; Kcomt et al., 2020; Sevelius et al., 2014; Summers et al., 2021), unmet transition needs put participants’ lives on hold and formed a barrier to addressing other healthcare concerns, due to de-prioritization of healthcare and concerns about experiencing discrimination in healthcare. Participants echoed a broader call from the TGD community to improve accessibility of GAMC by increasing capacity at gender clinics and implementing the ‘informed consent model’ of transgender care. Our findings additionally suggest that mTGD persons face unmet transition needs disproportionally often due to migrant-specific barriers to GAMC, stressing the need for equitable GAMC access for mTGD persons. For example, by allowing migrants to continue HRT which they previously started (formally or informally) without re-assessment for gender incongruence.

Consistent with prior qualitative studies among TGD persons (Gieles et al., 2023; Hostetter et al., 2022; Jaffee et al., 2016), having to educate providers outside gender-specialist settings about TGD healthcare placed a mental burden on participants. On top of this, we identified migrant-related barriers to bridging providers’ knowledge gaps, like newcomers’ unfamiliarity with the Dutch healthcare system, language barriers, and culturally influenced hesitancy to correct providers. We thus advise that, when implementing efforts to educate healthcare providers on TGD healthcare, special attention is payed to providers working in facilities that are frequented by migrants. It is nonetheless important to acknowledge that GPs are meant to have wide-ranging knowledge, rather than specialized knowledge, of medical topics. The solution to patients’ frustration about GP’s knowledge gaps regarding TGD-related healthcare therefore also lies in better communication about the role of GPs to patients, and improved capacity in gender-specialists centers to allow for referrals.

Our participants furthermore echoed prior studies which describe how seeking healthcare requires emotional labor for TGD persons, as they must combat the anticipation and sometimes the reality of unpleasant occurrences like misgendering or negative responses to identity disclosure (Gieles et al., 2023; Guss et al., 2019; Howard et al., 2019; Sherman et al., 2022). Our findings suggest this emotional toll may be especially high for mTGD individuals, as they are vulnerable to othering and discrimination on multiple axes - namely, gender, sexuality, ethnicity, skin color, legal status, and relevant language skills (Howard et al., 2019; Kattari et al., 2015, 2020; Sherman et al., 2022) - and often have a personal history of exclusion, discrimination, or trauma; many participants in this study left their country of origin at least in part because of gender-based discrimination and violence. Taking a proactive stance in creating a safe healthcare environment is thus crucial to engage mTGD people. For example, by displaying kindness and empathy, being supportive of a patients’ autonomy over their gender identity, practicing cultural competency, and practicing trauma-informed care. Notably, multiple participants voiced a preference for fellow trans or queer providers in order to feel safe. Opportunities for such ‘concordant care’ (i.e. where patient and provider share demographic characteristics) have mainly been described in the context of ethnicity (Shen et al., 2018), although some qualitative studies among BIPOC TGD persons also described patients’ preference for concordance regarding gender identity (Howard et al., 2019; Sherman et al., 2022). Approaches to respond to this desire for concordance could include improving trans, migrant, and cultural representation on all levels of healthcare (for example, by ensuring a safe working environment for (m)TGD staff in combination with diversity hiring initiatives). Peer-led and community-based healthcare settings also provide concordance, but may inadvertently isolate TGD persons into a separate healthcare system and impose additional burdens on an already strained healthcare system.

The number of participants who mentioned precarious living circumstances was markedly high among our participants. This mirrors prior reports from Europe and the United States which describe higher rates of poverty, discrimination, and mental health issues among ethnic minority or migrant TGD persons, as compared to ethnic majority or non-migrant TGD individuals (Grant et al., 2011; James et al., 2016; Russell et al., 2019). In addition to forming practical barriers to healthcare (like being unable to cover healthcare costs), this also resulted in de-prioritization of healthcare. Like in previous studies (Remien et al., 2015), some participants postponed healthcare until they found stable housing and work. Offering integrated care (including somatic, mental, social and legal care) may thus be especially beneficial for mTGD individuals with multifaceted health and social needs. Integrated care can be provided under-one-roof, or by strengthening the ties and communication between care organizations.

Many participants had difficulty finding information on healthcare in general and GAMC specifically, because online information was dispersed and often Dutch-only. This illustrates the importance of materials that explain, in multiple languages, the basics and specifics of finding (TGD-related) healthcare in the Netherlands. For example, Queers Beyond Borders provide a comprehensive healthcare and social map for queer migrants in a number of European cities (https://www.queersbeyondborders.info). The finding that having a social network of peers was a main facilitator to health system literacy also notes an opportunity to improve healthcare access for mTGD individuals by setting up LGBTQI+ community spaces and events. Spaces and events that aim to engage LGBTQI+ refugees are especially important, since refugee centers are often located far from LGBTQI+ community hubs. Prior studies endorse the importance of a social support network as facilitator to healthcare access (Ross et al., 2023).

Overall, our findings highlight an opportunity to improve healthcare access for mTGD by offering education about TGD persons and TGD-related health(care) to healthcare workers, especially those based in refugee centers and other migrant-frequented settings. Education can focus on the following three aspects. First, on addressing the beliefs, myths and stereotypes that healthcare staff may have about TGD persons. This can help to decrease the incidence of perceived discrimination and has been shown to increase providers’ willingness to learn about TGD-specific healthcare needs (Stroumsa et al., 2019). Second, on TGD cultural competence (e.g. exchanging pronouns, navigating uncertainty, and handling mistakes) and migrant cultural competence (e.g. reflecting on your cultural bias, assessing a patient’s health literacy and expectations, and being aware of possible trauma). This could facilitate safe patient-provider relationships. Existing guides on cultural competence, developed in collaboration with healthcare workers and the community, can be used for this (Braybrook et al., 2022; World Health Organization, 2021). Third, on TGD health and healthcare, including triage (i.e. which issues require gender-specialist care) and the TGD healthcare landscape (including community resources like support groups and service organizations). This can help to reduce the incidence of mismatched care responses and unnecessary referrals. Seminars about these topics can be helpful for interested individual providers. However, including these topics in medical curricula of diverse health studies and specializations is needed to achieve more systemic changes in provider knowledge about migrant- and TGD related health (van Heesewijk et al., 2022).

Finally, we acknowledge that there is no universal experience of “the migrant transgender individual” when trying to access healthcare. Experiences will inevitably be shaped by several factors including one’s migration history (e.g. forced versus unforced, age at migration, and years since migration), current legal and socioeconomic status, mental health, personality, relevant language skills, and transition journey.

### Strength and limitations

A strength of this study was its focus on mTGD individuals, who form a subpopulation of TGD individuals with specific intersectional challenges that are often overlooked. Furthermore, our research team included members of the migrant and BIPOC TGD community, who contributed to developing the interview guide, recruiting participants, conducting interviews, the interpretation of findings, and dissemination of findings. This community-involvement could explain the sizeable number of included participants (*n* = 32) and the openness with which interviewers were met. We managed to include a diverse sample mTGD participants, born in 27 different countries from seven world regions, and with variations in migration history, residence status, and health insurance.

This study is not without limitations. First, we did not explore barriers to healthcare among mTGD people without Dutch or English language skills. We assume that this would result in even greater barriers to healthcare. Second, recruiting participants through the Amsterdam Trans Clinic may have resulted in selection bias. This population includes individuals with the circumstances, skills, or network needed to find this clinic, and individuals with positive attitudes toward community-based healthcare. On the other hand, the clinic includes a subpopulation of mTGD who are particularly marginalized. Third, there were limitations to our CBPR approach. The research question did not fully arise from the mTGD community itself, but was formulated by a non-migrant transgender researcher working at the Amsterdam Trans Clinic. Thematic coding was conducted by one cisgender and one transgender team member, both of whom are white and non-migrant; this manuscript was first drafted by a cisgender, white, non-migrant team member. Although mTGD community researchers were involved in defining themes and provided feedback on this manuscript, cisnormative and ethnocentric ideas or phrasings may have persisted. Pre-planned financial reimbursements for community researchers were insufficient to adequately cover the time spent on manuscript feedback after data collection was completed. In future endeavors, employment contracts should ensure that no researcher does any unpaid work throughout the research process.

### Conclusion

To conclude, mTGD individuals face unique barriers to healthcare owing to their intersecting migrant and gender identities. The main barriers to healthcare were provider knowledge gaps, experiences of identity invalidation and discrimination on multiple axes, health system factors like conservative referral policies and lacking continuity of care, and the prevalence of precarious living circumstances. Access to healthcare for mTGD can be improved by enhancing provider awareness of these barriers, knowledge on TGD healthcare and cultural competency among providers (especially those who serve migrants). Ensuring equitable access to GAMC for mTGD or integrating GAMC with other healthcare disciplines and social support is advised, as mTGD with unmet GAMC needs may de-prioritize other healthcare. The findings of this study underscore the call for trans-affirming and culturally inclusive healthcare environments so that mTGD people feel safe, understood, and supported when seeking care.

## Data Availability

The participants of this study did not give written consent for their data to be shared publicly. Due to the sensitive nature of the research, supporting data is not available.

## Appendix A to manuscript entitled “the intersection between transgender identity and migrant background: experienced barriers and facilitators to healthcare in The Netherlands”

### Interview guide

**00. Opening the interview:**

- Can you tell me how you came to the Amsterdam Trans Clinic? (why did you decide to come to the Trans Clinic and how did you go find it?)

**01. Barriers and facilitators to accessing healthcare**

- What sort of health care have you needed?
- How did you go about finding this care? (For example, did you look for information online, or did you consult friends, or your GP?)
- Were there any obstacles that you encountered when trying to access this care? Could you tell me a bit more about this obstacle…
  - In what way did those factors make it more difficult to access healthcare?
  - What did you do when you encountered this obstacle? (e.g., ways to overcome obstacles, trying to find care elsewhere?)
  - Did you manage to access healthcare? If not, how did you deal with this?
  - How do you think these difficulties can be overcome or prevented?
- Were there any factors that helped you to access this care?
  - In what way did those factors make it easier to access healthcare?

Here, it is possible to prompt for more specific topics, for example:

- Did you experience obstacles relating to the health system, costs or documentation?
- How did you experience the relation between you and your doctor?
- Did you receive support from people around you? Did that influence your ability to find care (and if so, how)?
- Do you feel your personal life had any influence on your ability to access care? If so, how?

**02. Experiences in healthcare**

- If you managed to access the care you needed, what was your experience like?
  - What were the reasons that your experience was positive/negative?
    For example, any things that your provider did, or factors relating to the healthcare environment?
- How did you experience the relationship between you and your doctor?

#### Asking about healthcare not previously mentioned

- Have you needed sexual health care?
  - Have you wanted to get tested for STI or HIV, and if so, have you gotten STI/HIV testing?
  - Have you wanted to use PrEP for HIV prevention, and if so, have you gotten PrEP care?
  - Prompt questions from Part 01-02
- Have you needed or wanted mental/psychosocial healthcare?
  - Prompt questions from Part 01-02
- Have you needed care for complaints or diseases of the body?
  - Prompt questions from Part 01-02
- Have you needed gender-affirming health services?
  - Prompt questions from Part 01-02

**04. Hypothetical future scenarios** (e.g., if a participant says to not have needed healthcare (yet))

- Would you visit a doctor if you had an urgent health issue?
  - If not, why not?
  - If yes, how would you go about finding that care? Do you anticipate obstacles?
- Would you visit a doctor if you had a non-urgent health issue?
  - If not, why not?
  - If yes, how would you go about finding that care? Do you anticipate obstacles?

**05. Overall suggestions**

- Overall, how do you think we can make health care more accessible for you?

## Appendix B: Questionnaire form

To be answered by interviewee:

1. What is your age (years)? ….
2. What is your gender identity?
  □  (Trans) man
  □  (Trans) woman
  □ Neither male nor female
  □ Both male and female
  □ I don’t know
  □ Other: (please describe)
  …………………………
3. What are your pronouns?
  □ He/him
  □ She/her
  □ They/them
  □ other: (please describe)
  …………………………
4. What is the sex you were assigned at birth?
  □ Male
  □ Female
  □ Other: (please describe)
  …………………………
5. In which country were you born?
  …………………………….
6. In which country were your parents born?
  Mother: ……………………
  Father: …………………….
7. What is your first language?
  …………………………….
8. Since when have you been in the Netherlands? (day, month, year?)
  …………………………….
9. What is your highest level of education?
  …………………………….
10. What is your current residence status in the Netherlands?
  □ Dutch citizenship (Dutch passport/ID)
  □ European citizenship (EU passport/ID)
  □ 5-year Residence Permit
  □ Refugee status / Asylum procedure
  □ Undocumented
  □ Other: (please describe)
  …………………………….
11. What is your current housing situation?
  □ Independent housing
  □ Shared housing
  □ Staying with family/friend/acquaintance
  □ Homeless, incl. homeless shelter
  □ Asylum seekers’ accommodation
  □ Supported living / institution
  □ Other: (please describe)
  …………………………
12. Do you have health insurance?
  □ Dutch health insurance
  □ Insurance for asylum seekers
  □ Insured abroad
  □ No insurance at all
  □ Other way to pay for health care:
  …………………………
13. What are your current forms of income / access to personal finances? (multiple answers possible)
  □ Formal Work (please describe)
  …………………………
  □ Informal Work
  …………………………
  □ Government support
  …………………………
  □ Financial support by others (family, partner, friends, acquaintances)
  …………………………
  Other (please describe)
  …………………………

## References

[CIT0001] Brandenberger, J., Tylleskär, T., Sontag, K., Peterhans, B., & Ritz, N. (2019). A systematic literature review of reported challenges in health care delivery to migrants and refugees in high-income countries – the 3C model. *BMC Public Health*, *19*(1), 755. 10.1186/s12889-019-7049-x 31200684 PMC6567460

[CIT0002] Braun, V., & Clarke, V. (2006). Using thematic analysis in psychology. *Qualitative Research in Psychology*, *3*(2), 77–101. 10.1191/1478088706qp063oa

[CIT0003] Braveman, P. (2014). What are health disparities and health equity? We need to be clear. *Public Health Reports (Washington, D.C.: 1974)*, *129*(Suppl 2), 5–8. 10.1177/00333549141291S203 PMC3863701 24385658

[CIT0004] Braybrook, D., Bristowe, K., Timmins, L., Roach, A., Day, E., Clift, P., Rose, R., Marshall, S., Johnson, K., Sleeman, K., & Harding, R. (2022). *ABC of LGBT+ inclusive communication: A guide for health and social care professionals*. ACCESS Care.

[CIT0005] Central Committee on Research Involving Human Subjects (CCMO). (2024). Legal Framework for Medical Scientific Research https://www.ccmo.nl/onderzoekers/aanvullende-informatie-over-bepaalde-soorten-onderzoek/niet-wmo-onderzoek/dossieronderzoek

[CIT0006] Coleman, E., Radix, A. E., Bouman, W. P., Brown, G. R., de Vries, A. L. C., Deutsch, M. B., Ettner, R., Fraser, L., Goodman, M., Green, J., Hancock, A. B., Johnson, T. W., Karasic, D. H., Knudson, G. A., Leibowitz, S. F., Meyer-Bahlburg, H. F. L., Monstrey, S. J., Motmans, J., Nahata, L., … Arcelus, J. (2022). Standards of Care for the Health of Transgender and Gender Diverse People, Version 8. *International Journal of Transgender Health*, *23*(Suppl 1), S1–S259. 10.1080/26895269.2022.2100644 36238954 PMC9553112

[CIT0007] Connolly, D., & Gilchrist, G. (2020) Prevalence and correlates of substance use among transgender adults: A systematic review. *Addictive Behaviors*, *111*, 106544. 10.1016/j.addbeh.2020.106544 32717497

[CIT0008] Gerritse, K., Hartman, L. A., Bremmer, M. A., Kreukels, B. P. C., & Molewijk, B. C. (2021).) Decision-making approaches in transgender healthcare: Conceptual analysis and ethical implications. *Medicine, Health Care, and Philosophy*, *24*(4), 687–699. 10.1007/s11019-021-10023-6 34008081 PMC8557156

[CIT0009] Gieles, N. C., Zinsmeister, M., Pulles, S., Harleman, A., van Heesewijk, J., & Muntinga, M. (2023). The medical world is very good at cis people, but trans is a specialisation’. Experiences of transgender and non-binary people with accessing primary sexual and reproductive healthcare services in the Netherlands. *Glob Public Health*, *18*(1), 2246059. 10.1080/17441692.2023.2246059 37585600

[CIT0010] Government of the Netherlands (2018). LGBTQ equality in the Netherlands. Retrieved 28 March 2024 from https://www.government.nl/binaries/government/documenten/leaflets/2018/06/01/lgbti-equality-in-the-netherlands/180718+Factsheet+NETH_LGBTI+Equality.pdf

[CIT0011] Grant, J. M., Mottet, L. A., Tanis, J., Harrison, J., Herman, J. L., & Keisling, M. (2011). *National transgender discrimination survey report on health and health care*. National Center for Transgender Equality and National Gay and Lesbian Task Force.

[CIT0012] Green, J., & Thorogood, N. (2018). *Qualitative methods for health research*. SAGE Publications Ltd.

[CIT0013] Guss, C. E., Woolverton, G. A., Borus, J., Austin, S. B., Reisner, S. L., & Katz-Wise, S. L. (2019). Transgender adolescents’ experiences in primary care: A qualitative study. *The Journal of Adolescent Health: Official Publication of the Society for Adolescent Medicine*, *65*(3), 344–349. 10.1016/j.jadohealth.2019.03.009 31227384 PMC6708717

[CIT0014] Hamed, S., Bradby, H., Ahlberg, B. M., & Thapar-Björkert, S. (2022). Racism in healthcare: A scoping review. *BMC Public Health*, *22*(1), 988. 10.1186/s12889-022-13122-y 35578322 PMC9112453

[CIT0015] Hostetter, C. R., Call, J., Gerke, D. R., Holloway, B. T., Walls, N. E., & Greenfield, J. C. (2022). We are doing the absolute most that we can, and no one is listening": Barriers and facilitators to health literacy within transgender and nonbinary communities. *International Journal of Environmental Research and Public Health*, *19*(3), 1229. 10.3390/ijerph19031229 35162254 PMC8834767

[CIT0016] Howard, S. D., Lee, K. L., Nathan, A. G., Wenger, H. C., Chin, M. H., & Cook, S. C. (2019). Healthcare experiences of transgender people of color. *Journal of General Internal Medicine*, *34*(10), 2068–2074. 10.1007/s11606-019-05179-0 31385209 PMC6816758

[CIT0017] Israel, B. A., Schulz, A. J., Parker, E. A., & Becker, A. B. (1998). Review of community-based research: Assessing partnership approaches to improve public health. *Annual Review of Public Health*, *19*(1), 173–202. 10.1146/annurev.publhealth.19.1.173 9611617

[CIT0018] Jaffee, K. D., Shires, D. A., & Stroumsa, D. (2016).) Discrimination and delayed health care among transgender women and men: Implications for improving medical education and health care delivery. *Medical Care*, *54*(11), 1010–1016. 10.1097/MLR.0000000000000583 27314263

[CIT0019] James, S. E., Herman, J. L., Durso, L. E., & Heng-Lehtinen, R. (2024). *Early Insights: A Report of the 2022 U.S. Transgender Survey*. National Center for Transgender Equality.

[CIT0020] James, S. E., Herman, J. L., Rankin, S., Keilsing, M., Mottet, L., & Anafi, M. (2016). *The Report of the 2015 U.S. Transgender Survey*. National Center for Transgender Equality.

[CIT0021] Kattari, S. K., Bakko, M., Hecht, H. K., & Kinney, M. K. (2020).) Intersecting experiences of healthcare denials among transgender and nonbinary patients. *American Journal of Preventive Medicine*, *58*(4), 506–513. 10.1016/j.amepre.2019.11.014 32001054

[CIT0022] Kattari, S. K., W, N., Whitfield, D. L., & Langenderfer-Magruder, L. (2015). Racial and ethnic differences in experiences of discrimination in accessing health services among transgender people in the United States. *International Journal of Transgenderism*, *16*(2), 68–79. 10.1080/15532739.2015.1064336

[CIT0023] Kcomt, L., Gorey, K. M., Barrett, B. J., & McCabe, S. E. (2020).) Healthcare avoidance due to anticipated discrimination among transgender people: A call to create trans-affirmative environments. *SSM - Population Health*, *11*, 100608. 10.1016/j.ssmph.2020.100608 32529022 PMC7276492

[CIT0024] Kennisinstituut van de Federatie Medisch Specialisten (2024). Evaluatie van de kwaliteitsstandaard Transgenderzorg - somatisch [Dutch]. https://www.rijksoverheid.nl/documenten/rapporten/2024/04/05/evaluatierapport-transgenderzorg-somatisch-kennisinstituut-fms

[CIT0025] Khanom, A., Alanazy, W., Couzens, L., Evans, B. A., Fagan, L., Fogarty, R., John, A., Khan, T., Kingston, M. R., Moyo, S., Porter, A., Rhydderch, M., Richardson, G., Rungua, G., Russell, I., & Snooks, H. (2021). Asylum seekers’ and refugees’ experiences of accessing health care: A qualitative study. *BJGP Open*, *5*(6), BJGPO.2021.0059. 10.3399/BJGPO.2021.0059 34376383 PMC9447303

[CIT0026] Kommattam, P., Verkerke, V., Verdonk, J., Huges, S., van Diemen, A., Berghouwer, B., Bastaans, S. (2017). Trans healthcare in the Netherlands. https://principle17.org/sites/default/files/Report%20P17%20trans%20health%20care%20Netherlands.pdf

[CIT0027] Leone, A. G., Trapani, D., Schabath, M. B., Safer, J. D., Scout, N. F. N., Lambertini, M., Berardi, R., Marsoni, S., Perrone, F., Cinieri, S., Miceli, R., Morano, F., & Pietrantonio, F. (2023). Cancer in transgender and gender-diverse persons: A review. *JAMA Oncology*, *9*(4), 556–563. 10.1001/jamaoncol.2022.7173 36757703

[CIT0028] Pinna, F., Paribello, P., Somaini, G., Corona, A., Ventriglio, A., Corrias, C., Frau, I., Murgia, R., El Kacemi, S., Galeazzi, G. M., Mirandola, M., Amaddeo, F., Crapanzano, A., Converti, M., Piras, P., Suprani, F., Manchia, M., Fiorillo, A., & Carpiniello, B; Italian Working Group on, L. M. H. (2022). Mental health in transgender individuals: A systematic review. *International Review of Psychiatry (Abingdon, England)*, *34*(3–4), 292–359. 10.1080/09540261.2022.2093629 36151828

[CIT0029] Remien, R. H., Bauman, L. J., Mantell, J. E., Tsoi, B., Lopez-Rios, J., Chhabra, R., DiCarlo, A., Watnick, D., Rivera, A., Teitelman, N., Cutler, B., & Warne, P. (2015). Barriers and facilitators to engagement of vulnerable populations in HIV primary care in New York City. *Journal of Acquired Immune Deficiency Syndromes (1999)*, *69* (1), S16–S24. 10.1097/QAI.0000000000000577 25867774 PMC4559146

[CIT0030] Ross, M. B., van de Grift, T. C., Elaut, E., Nieder, T. O., Becker-Hebly, I., Heylens, G., & Kreukels, B. P. C. (2023). Experienced barriers of care within European treatment seeking transgender individuals: A multicenter ENIGI follow-up study. *International Journal of Transgender Health*, *24*(1), 26–37. 10.1080/26895269.2021.1964409 36713146 PMC9879197

[CIT0031] Russell, C., Sanders, F., Watkins, F. (2019). Intersectional analysis from findings of the European Union Agency for Fundamental Rights (FRA) 2nd LGBTI survey on LGBTI people in the EU and North Macedonia and Serbia. Retrieved 11 July 2024 from https://www.ilga-europe.org/report/intersections-trans-non-binary-diving-into-the-fra-lgbti-ii-survey-data/

[CIT0032] Sevelius, J. M., Patouhas, E., Keatley, J. G., & Johnson, M. O. (2014).) Barriers and facilitators to engagement and retention in care among transgender women living with human immunodeficiency virus. *Annals of Behavioral Medicine: A Publication of the Society of Behavioral Medicine*, *47*(1), 5–16. 10.1007/s12160-013-9565-8 24317955 PMC3925767

[CIT0033] Shen, M. J., Peterson, E. B., Costas-Muñiz, R., Hernandez, M. H., Jewell, S. T., Matsoukas, K., & Bylund, C. L. (2018).) The effects of race and racial concordance on patient-physician communication: A systematic review of the literature. *Journal of Racial and Ethnic Health Disparities*, *5*(1), 117–140. 10.1007/s40615-017-0350-4 28275996 PMC5591056

[CIT0034] Sherman, A. D. F., Balthazar, M. S., Daniel, G., Bonds Johnson, K., Klepper, M., Clark, K. D., Baguso, G. N., Cicero, E., Allure, K., Wharton, W., & Poteat, T. (2022). Barriers to accessing and engaging in healthcare as potential modifiers in the association between polyvictimization and mental health among Black transgender women. *PloS One*, *17*(6), e0269776. 10.1371/journal.pone.0269776 35709158 PMC9202936

[CIT0035] Sociaal Cultureel Planbureau (2017). Transgender personen in Nederland. Retrieved 28 March 2024 from https://www.scp.nl/binaries/scp/documenten/publicaties/2017/05/09/transgender-personen-in-nederland/Transgender+personen+in+Nederland.pdf

[CIT0036] Streed, C. G., Beach, L. B., Caceres, B. A., Dowshen, N. L., Moreau, K. L., Mukherjee, M., Poteat, T., Radix, A., Reisner, S. L., & Singh, V; American Heart Association Council on Peripheral Vascular, D., Council on Arteriosclerosis, T., Vascular, B., Council on, C., Stroke, N., Council on Cardiovascular, R., Intervention, Council on, H., & Stroke, C (2021). Assessing and addressing cardiovascular health in people who are transgender and gender diverse: A scientific statement from the American Heart Association. *Circulation*, *144*(6), e136–e148. 10.1161/CIR.0000000000001003 34235936 PMC8638087

[CIT0037] Stroumsa, D., Shires, D. A., Richardson, C. R., Jaffee, K. D., & Woodford, M. R. (2019).) Transphobia rather than education predicts provider knowledge of transgender health care. *Medical Education*, *53*(4), 398–407. 10.1111/medu.13796 30666699

[CIT0038] Summers, N. A., Huynh, T. T., Dunn, R. C., Cross, S. L., & Fuchs, C. J. (2021).) Effects of gender-affirming hormone therapy on progression along the HIV care continuum in transgender women. *Open Forum Infectious Diseases*, *8*(9), ofab404. 10.1093/ofid/ofab404 34514019 PMC8415531

[CIT0039] van der Loos, M., Hannema, S. E., Klink, D. T., den Heijer, M., & Wiepjes, C. M. (2022).) Continuation of gender-affirming hormones in transgender people starting puberty suppression in adolescence: A cohort study in the Netherlands. *The Lancet. Child & Adolescent Health*, *6*(12), 869–875. 10.1016/S2352-4642(22)00254-1 36273487

[CIT0040] Van Gerwen, O. T., Jani, A., Long, D. M., Austin, E. L., Musgrove, K., & Muzny, C. A. (2020). Prevalence of sexually transmitted infections and human immunodeficiency virus in transgender persons: A systematic review. *Transgender Health*, *5*(2), 90–103. 10.1089/trgh.2019.0053 32656353 PMC7347015

[CIT0041] van Heesewijk, J., Kent, A., van de Grift, T. C., Harleman, A., & Muntinga, M. (2022). Transgender health content in medical education: A theory-guided systematic review of current training practices and implementation barriers & facilitators. *Advances in Health Sciences Education: Theory and Practice*, *27*(3), 817–846. 10.1007/s10459-022-10112-y 35412095 PMC9374605

[CIT0042] Wall, C. S. J., Patev, A. J., & Benotsch, E. G. (2023). Trans broken arm syndrome: A mixed-methods exploration of gender-related medical misattribution and invasive questioning. *Social Science & Medicine (1982)*, *320*, 115748. 10.1016/j.socscimed.2023.115748 36736052

[CIT0043] Wiepjes, C. M., Nota, N. M., de Blok, C. J. M., Klaver, M., de Vries, A. L. C., Wensing-Kruger, S. A., de Jongh, R. T., Bouman, M. B., Steensma, T. D., Cohen-Kettenis, P., Gooren, L. J. G., Kreukels, B. P. C., & den Heijer, M. (2018). The Amsterdam cohort of gender dysphoria study (1972–2015): Trends in prevalence, treatment, and regrets. *The Journal of Sexual Medicine*, *15*(4), 582–590. 10.1016/j.jsxm.2018.01.016 29463477

[CIT0044] Winter, S., Diamond, M., Green, J., Karasic, D., Reed, T., Whittle, S., & Wylie, K. (2016). Transgender people: Health at the margins of society. *Lancet (London, England)*, *388*(10042), 390–400. 10.1016/S0140-6736(16)00683-8 27323925

[CIT0045] World Health Organization (2021). *Refugee and migrant health: Global competency standards for health workers*. Retrieved 11 July 2024 from https://www.who.int/publications/i/item/9789240030626

